# A Prediction Model for High Risk of Positive RT-PCR Test Results in COVID-19 Patients Discharged From Wuhan Leishenshan Hospital, China

**DOI:** 10.3389/fpubh.2021.778539

**Published:** 2021-11-08

**Authors:** Yawei Qian, Guang Zeng, Yue Pan, Yang Liu, Limao Zhang, Kun Li

**Affiliations:** ^1^Department of Hepatobiliary and Pancreatic Surgery, Zhongnan Hospital of Wuhan University, Wuhan, China; ^2^Leishenshan Hospital, Wuhan, China; ^3^Department of General Surgery, The First Affiliated Hospital of Nanjing Medical University, Nanjing, China; ^4^Department of Urology, Zhongnan Hospital of Wuhan University, Wuhan, China; ^5^School of Naval Architecture, Ocean & Civil Engineering, Shanghai Jiao Tong University, Shanghai, China; ^6^School of Economics and Management, Wuhan University, Wuhan, China; ^7^Zhongnan Hospital of Wuhan University, Wuhan, China; ^8^School of Civil and Environmental Engineering, Nanyang Technological University, Singapore, Singapore

**Keywords:** COVID-19, recovered, patients, nucleic acid, re-detectable, predict, model

## Abstract

Several recent studies have reported that a few patients had positive SARS-CoV-2 RNA tests after hospital discharge. The high-risk factors associated with these patients remain to be identified. A total of 463 patients with COVID-19 discharged from Leishenshan Hospital in Wuhan, China, between February 8 and March 8, 2020 were initially enrolled, and 351 patients with at least 2 weeks of follow-up were finally included. Seventeen of the 351 discharged patients had positive tests for SARS-CoV-2 RNA. Based on clinical characteristics and mathematical modeling, patients with shorter hospital stays and less oxygen desaturation were at higher risk of SARS-CoV-2 RNA reoccurrence after discharge. Notably, traditional Chinese medicine treatment offered extensive benefits to reduce risk. Particular attention should be paid to those patients with high risk, and traditional Chinese medicine should be advocated.

## Introduction

In December 2019, patients with an unknown pneumonia, now called coronavirus disease 2019 (COVID-19), were first identified in Wuhan, China (1–3). As of April 3, 2020, mainland China had reported 82,857 confirmed cases of COVID-19. A total of 76,810 patients have recovered in accordance with the following criteria for hospital discharge: (a) body temperature returned to normal for more than 3 days; (b) respiratory symptoms improved significantly; (c) acute exudative lesions absorbed remarkably on chest images; and (d) two consecutive negative nucleic acid tests of respiratory tract specimens, including sputum and nasopharyngeal swabs, at a sampling interval of at least 24 h.

Recently, a few studies have indicated that a small number of recovered patients with COVID-19 had positive SARS-CoV-2 RNA tests (4, 5). However, it is still unclear how many discharged people have true positive nucleic acid test results. In addition, patients with a high risk of positive RT-PCR results after hospital discharge are difficult to differentiate. In this study, patients with confirmed COVID-19 from Leishenshan Hospital in Wuhan, China, who met the hospital discharge criteria were followed up for at least 2 weeks. We aimed to investigate the real rate of positive RT-PCR test results in recovered patients and establish an exact model of predicting repositive status in discharged patients.

## Methods

### Study Design and Participants

All recovered patients with COVID-19 from Leishenshan Hospital in Wuhan, China, who met the criteria for hospital discharge were enrolled between February 8 and March 8. Leishenshan hospital was one of the makeshift hospitals used to treat and isolate patients infected with SARS-CoV-2; it was rapidly built in 1 week and was entrusted by Zhongnan Hospital of Wuhan University, China. A total of 453 discharged patients were initially included and were monitored up to March 22, 2020, the final date of follow-up. Among them, 42 patients were lost to follow-up, and five patients were excluded for missing RT-PCR results after hospital discharge. The detailed medical records of the remaining 406 patients were retrospectively screened, and 55 patients were excluded for missing core data, including highest temperature, lowest oxygen saturation and routine blood examination. A total of 351 discharged patients were finally included in this study. The study was approved by the ethics committee of Zhongnan Hospital of Wuhan University (No. 2020074). The informed consent of the patients was waived for this infectious disease by the ethics committee.

### Data Collection

Of the initially included 406 patients, medical records including clinical characteristics and laboratory indices were collected and reviewed. Among 406 discharged patients, the SARS-CoV-2 RNA test results of 19 patients were repositive, and the detailed follow-up information is described in Table 1. The majority of the clinical data used in this study were collected on admission unless otherwise indicated. Patients were classified according to the guidelines for the diagnosis and treatment of COVID-19 (7th trial edition). The highest temperature was considered the highest body temperature from illness onset to hospital discharge from Leishenshan Hospital in Wuhan, China. According to chest radiography findings, the severity of pulmonary lesions was classified as normal, mild, moderate, or severe based on whether lesions were not present or involved a unilateral lobe, multiple lobes in both lungs, or all lobes in both lungs, respectively.

**Table 1:** The detailed follow-up information of 19 discharged patients with positive RT-PCR results.

**ID**	**Type**	**Symptom**	**Serum antibody**			**Chest CT**	**Days since hospital dicharge to first positive RNA**	**Therapy**
			**Total**	**IgM**	**IgG**			
1	Common	None	NA	Negative	Negative	NA	11	Convalescent plasma therapy
2	Common	None	Positive	NA	NA	Light	10	Oxygen therapy
3	Common	None	NA	NA	NA	Light	6	None
4	Common	None	NA	NA	NA	NA	11	None
5	Common	None	NA	Positive	Positive	Light	9	Antiviral therapy
6	Common	None	NA	NA	NA	NA	10	None
7	Common	Chest distress	NA	Weakly positive	Positive	Light	10	Antiviral therapy
8	Mild	None	NA	NA	NA	NA	7	None
9	Mild	None	NA	Weakly positive	Positive	Normal	5	Antiviral therapy
10	Mild	None	NA	NA	NA	NA	10	None
11	Severe	None	NA	Positive	Positive	NA	10	Antiviral therapy
12	Mild	None	NA	Negative	Positive	NA	5	None
13	Mild	None	Positive	NA	NA	NA	8	Antiviral therapy
14	Mild	Chest distress	NA	NA	NA	NA	9	None
15	Common	None	Positive	NA	NA	Normal	10	None
16	Common	None	NA	Positive	Positive	NA	14	None
17	Mild	None	NA	NA	NA	NA	12	None
18	Common	None	NA	Weakly positive	Positive	Light	10	Antiviral therapy
19	Severe	None	NA	NA	NA	NA	11	None

### Data for Random Forest Analysis

The proposed method was applied to a real dataset aiming to predict patients' physical condition after discharge. Each record contained 21 features with multiple types, including numeric, Boolean, and category, as outlined in Table 2. To train and deploy the random forest (RF) algorithm, each record needed to be labeled with 0 or 1. We set our classification target to identify high-risk patients with redetectable positive RT-PCR results or symptoms after hospital discharge and assigned the label 1 to them. Other patients who made a full recovery were labeled 0.

**Table 2:** Description of features in two classes.

**No**.	**Feature**	**Type**	**Label = 0 (*n* = 291)**	**Label =1(*n* = 60)**	***p***
1	Male sex, No. (%)	Boolean	135 (46.4)	23 (38.3)	0.11
2	Age, Mean [IQR]	Numerical	57.2 [46.0,69.0]	50.5 [37.3, 63.8]	0.21
3	Hospital stays, M [IQR]	Numerical	14.5 [11.0, 18.0]	14.1 [10.0, 17.8]	0.005
4	Course of disease	Numerical	14.4 [7.0, 20.0]	15.43 [10.0, 20.0]	0.23
5	Have history of contact, No (%)	Boolean	37 (12.7)	7 (11.7)	0.54
6	Have first fever, No (%)	Boolean	193 (66.3)	42 (70.0)	1.00
7	Have first breath, No (%)	Boolean	217 (74.6)	48 (80.0)	0.51
8	Have first digestion, No (%)	Boolean	25 (8.6)	6 (10.0)	0.42
9	Have first nervous, No (%)	Boolean	126 (43.3)	24 (40.0)	0.44
10	Have first others, No (%)	Boolean	7 (2.4)	8 (13.3)	1.00
11	Lowest blood oxygen, M [IQR]	Numerical	95.1[94.0, 96.0]	95.0 [95.0, 96.0]	0.97
12	Oxygen inhalation, No (%)	Categorical			0.72
	Normal		137 (47.1)	28 (46.7)	
	1–2 L		54 (18.6)	12 (20.0)	
	2–4 L		26 (8.9)	5 (8.3)	
	>4 L		73 (25.1)	13 (32.1)	
	Mask		1 (0.3)	2 (3.3)	
13	Under health conditons	Numerical	0.64 [0, 1]	0.85 [0, 1]	0.62
14	Clinical diagnosis case, No (%)	Boolean	145 (49.8)	30 (50.0)	0.50
15	Level	Categorical	85 (29.2)	19 (31.7)	0.007
	i		85 (29.2)	19 (31.7)	
	ii		176 (61.5)	33 (55.0)	
	iii		27 (9.3)	8 (13.3)	
	iv		3 (1.0)	0 (0.0)	
16	Have treat 1, No. (%)	Categorical	94 (32.3)	24 (40.0)	0.05
17	Have treat 2, No. (%)		167 (58.1)	44 (73.3)	0.77
18	Have treat 3, No. (%)		54 (18.6)	10 (16.7)	0.67
19	Have treat 4, No. (%)		274 (95.2)	59 (98.3)	1.00
20	Have treat 5, No. (%)		260 (89.3)	56 (93.3)	0.25
21	CT findings, No. (%)	Categorical			0.18
	Mild		16 (5.5)	8 (13.3)	
	Moderate		59 (20.3)	18 (30.0)	
	Severe		196 (67.4)	29 (48.3)	
	Normal		20 (6.9)	5 (8.3)	

To make the cleaned dataset understandable to machine learning algorithms, all categorical features were split into sub-features by their categories and then expressed as binary values, i.e., one-hot encoding. Then, to prepare the data for model training and testing, a random partition was applied to the cleaned dataset to produce two subsets, the training set (280 to 80%) and the test set (71 to 20%). Furthermore, a type of data augmentation method, SMOTE, was used to oversample the minority class (label 0). We set the number of nearest neighbors to six, aiming to find the six nearest neighbors of a randomly selected minority instance, and calculated the Euclidean distance between them in the feature space. As a result, 299 synthetic minority class samples (label 1) were created for model training; this number was equal to the number of samples in label 0. In the pursuit of a more robust classification for patients at low or high risk, these prepared data were fed into the RF model. Finally, several evaluation metrics, including accuracy, precision, recall, F1-score, and area under the curve (AUC), were exploited to assess the classification performance of the RF model.

#### Data Preprocessing

Data preprocessing involves three major parts, namely the data cleaning, data encoding, data splitting and, data oversampling. To begin with, since raw data with various noise will adversely influence the result reliability, it is of necessity to perform data cleaning to ensure the data quality and format. To deal with the missing value, the main kind of noise in this research, we simply remove the entire row containing the null. Before these cleaned data are fed into machine learning algorithms, categorical variables need to be converted into binary vectors by the one-hot encoding, and then the whole dataset will be partitioned randomly into two subsets named the training and test sets in a ratio of 80–20%. To be specific, the model learns on the training set to make it generalized in other data, while the test set is used to evaluate the model performance.

Notably, a great concern is that our dataset will suffer from data imbalance, where the number of positive samples is much fewer than negative samples. To address it, a common oversampling approach called Synthetic Minority Oversampling Technique (SMOTE) is carried out to balance out the dataset (6). In other words, SMOTE can synthesize more records of the minority class in a balanced manner, enabling the model to learn the decision boundary effectively. The implementation of SMOTE starts from taking a random sample *x* from the minority class and find its *k* nearest neighbors in the feature space. Then, one of the *k* nearest neighbors will be chosen for creating the synthetic new minority instances *x*_*new*_ according to Equation (1).


(1) $$\begin{array}{r} x_{new}=x+rand\left(0,1\right)\times \left(x^{\left(i\right)}-x\right) \end{array}$$

where *x*^(*i*)^ are one of the *k* nearest neighbors of the randomly selected point *x*.

#### Random Forest Implementation

**Figure 1:**
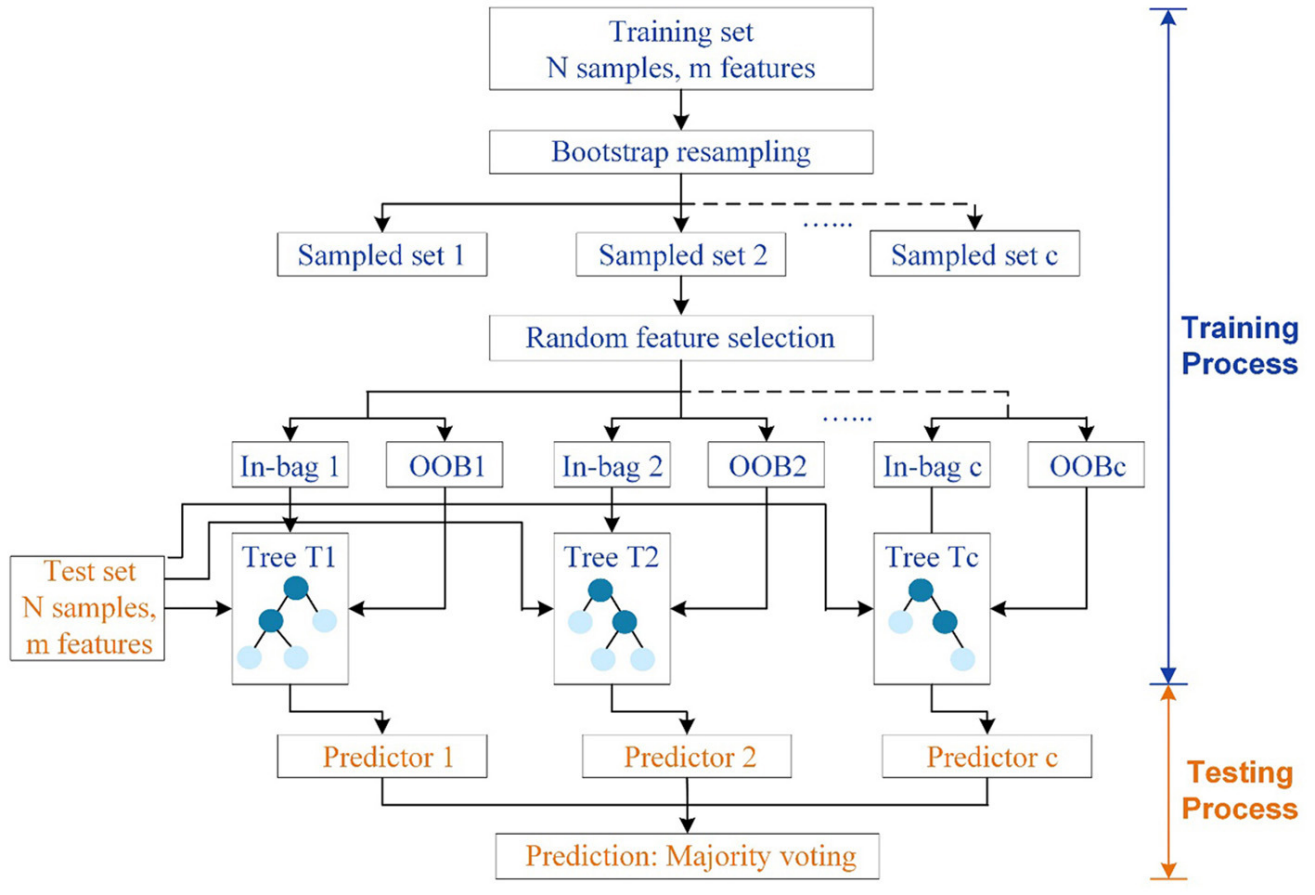
Structure of RF. The training set and test set have N samples in m features. C independent trees are generated for model fitting. Circles in dark blue denote tree nodes except leaves, while circles in light blue represent terminal nodes or leaves.

Motivated by the decision tree, which can be easily implemented and interpreted, RF is developed to combine multiple weak decision trees into a strong model in the pursue of a more robust final prediction. More specifically, RF as shown in Figure 1 is a kind of tree-based ensemble model to grow independent parallel estimators and aggregate their results under the idea of “Bagging” (7). There are two critical steps in training RF: one is the bootstrap re-sampling, and the other is random feature selection (8).

To construct RF with *C* decision trees *T*_1_(*X*), *T*_2_(*X*), …, *T*_*c*_(*X*) (where *X* represents features in an *m*-dimensional vector), a self-help method named bootstrap re-sampling technique should be firstly conducted to generate c different training set for *c* tree, which makes random sampling from *N* number of original training data with replacement. As a result, the size of the new and original training sets will be the same. Due to the sampling with replacement, some samples will be possibly used multiple times in a single tree, while some will not be selected for training trees during the bagging process. In other words, around 36.8% of the training data will be left out of the bootstrap sample, which is called out-of-bag (OOB) samples to serve as a validation set and estimate the generalization error. Subsequently, each bootstrapped training set is utilized for fully developing a decision tree with no additional pruning. Rather than relying on the whole features, subsets of features, which are randomly selected from all the *m* features, will be adopted to split nodes in each decision tree. Overall, these two steps will be repeated continuously until *C* distinct decision trees are built. The randomly generated forest containing diverse trees will return the classification result by taking the majority vote decision among the individual classifiers, which helps in reducing the variance and over fitting of the model (9). Also, RF has superior performance in high-dimensional data than other machine learning algorithms. Unlike black-box algorithms, RF can provide a pretty good indicator of the feature importance to examine the cause-effect behind the input and output variables.

Another significant advantage of RF is its powerful ability in quantifying feature importance based on the OOB data, which measures the drop of Gini impurity for a variable at each split point (10). Suppose that τ is a node of a decision tree T with *n* samples, the Gini impurity of node τ can be expressed as Equation (2). Then, the node can be split into two sub-nodes τ_*l*_ and τ_*r*_ by a certain variable *v*. Equation (3) quantifies the decrease of the Gini impurity at this split, which can also be defined as the Gini decrease for variable *v* at node τ. Finally, since other nodes can also be split by the variable *v*, the Gini decrease for variable *v* in all trees of the forest can be given as the summation in Equation (4), standing for the feature importance of *v*. The feature with a higher drop will contribute more to the classifier, which needs more concerns.


(2) $$\begin{array}{r} \Phi \left(\tau \right)=1-{\left(\frac{n_{0}}{n}\right)}^{2}-{\left(\frac{n_{1}}{n}\right)}^{2} \end{array}$$

(3) $$\begin{array}{r} \Delta \;\Phi \left(\tau \right)=\Phi \left(\tau \right)-\frac{n_{l}}{n}\Phi \left(\tau_{l}\right)-\frac{n_{r}}{n}\Phi \left(\tau_{r}\right) \end{array}$$

(4) $$\begin{array}{r} GI\left(v\right)=\underset{T}{\sum }\underset{\tau }{\sum }\Delta \;\Phi \left(\tau \right)I\left(\tau ,v\right) \end{array}$$

where *n*_0_ and *n*_1_ is the number of negative and positive samples, respectively. *n*_1_ and *n*_*r*_ is the number of the samples at two sub-nodes τ_*l*_ and τ_*r*_, respectively. *I*(τ, *v*) equals to one when *v* is the splitting variable of node τ, and 0 otherwise.

#### Statistical Evaluation

**Figure 2:**
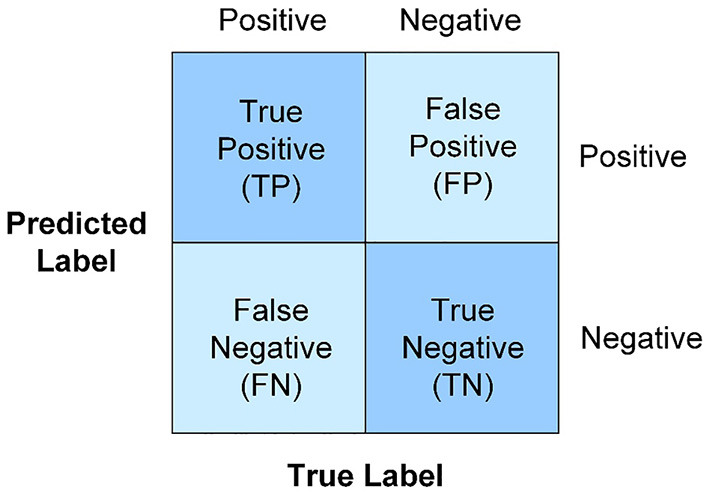
Confusion matrix. The accuracy, precision, recall, F1-score, and AUC mainly rely on the counts of instances correctly and incorrectly classified.


Metrics, named the accuracy, precision, recall, F1-score, and AUC mainly rely on the counts of instances correctly and incorrectly classified, which are tabulated in the confusion matrix in Figure 2. The number along the main diagonal refers to correct predictions, which can be divided by the total number of predictions to calculate accuracy, as defined in Equation (5). But accuracy is a poor measure for imbalanced data, when all instances tend to be predicted as the majority class to cause accuracy paradox. Thus, two additional metrics precision and recall in Equations (6) and (7) are also taken into account. Obviously, a low precision is caused by many false positive records, while a low recall comes from a greater number of false negative records. To jointly consider precision and recall, F1-score is expressed in the form of the harmonic mean in Equation (8). Besides, AUC is another metric to measure the overall model performance, which can be derived from the area under the receiver operating characteristics curve (ROC) about the relationship of the true positive rate and the false positive rate (FPR). All the above-mentioned metrics fall in the range of [0, 1]. The value closer to one implies better classification performance.

(5) $$\begin{array}{r} accuracy=\;\frac{TP+TN}{TP+FP+FN+TN} \end{array}$$

(6) $$\begin{array}{r} precision=\frac{TP}{TP+FP} \end{array}$$

(7) $$\begin{array}{r} recall=\;\frac{TP}{TP+FN} \end{array}$$

(8) $$\begin{array}{r} F1\;score=2\frac{precision\times recall}{precision+recall} \end{array}$$

where TP is the true positive class that is predicted positive, TN is the true negative class that is predicted negative, FP is the actual negative class that is predicted as positive. FN is the actual positive class that is predicted as negative.

### Statistical Analysis

Continuous data are presented as the mean ± SD, and categorical data are expressed as proportions. The quantitative variables were compared by Student's *t* test or Wilcoxon signed ranks test, when appropriate. Meanwhile, the categorical variables were analyzed using the Chi-square test or Fisher exact test. A two-sided *p* < 0.05 was considered statistically significant. These statistical analyses were performed with SPSS software.

## Results

### Demographics and Clinical Characteristics of 351 Discharged Patients

In this study, a total of 351 patients with COVID-19 (193 females and 158 males) were included, and 17 discharged patients had repositive RT-PCR tests. The characteristics of all patients are shown in Table 3. Among them, the median age was 56.2 years (range, 2–90 years), and 164 (46.7%) were more than 60 years old. Forty-Four patients had close contacted with relative diagnosed previously. The most commonly initial symptoms were respiratory symptoms (*n* = 266, 75.8%), fever (*n* = 235, 67.0%), neuromuscular (*n* = 150, 42.7%), digestive symptoms (*n* = 31, 8.8%), and others (*n* = 15, 4.3%), including catarrhus and conjunctivitis. In this study population, 175 (49.9%) patients were coexisted with one or more health conditions, such as hypertension, lung disease, and diabetes. The median time from symptom onset to admission was 14.6 ± 8.4 days, whereas 31 (8.8%) patients developed high fever (body temperature ≥39.0). In the entire cohort, 225 patients developed severe abnormal chest CT findings and the median level of lowest oxyhemoglobin saturation (%) was 95.1 ± 2.5, while the median numbers of white blood cell and absolute lymphocyte were within normal range. Of these 351 patients, 186 patients received oxygen inhalation using nasal catheter or mask. In addition, most (*n* = 287, 81.8%) accept Chinese medicine and drug treatment, whereas more than half (*n* = 233, 66.4%) of patients received antiviral therapy, including abidor hydrochloride capsules, oseltamivir phosphate capsules and lopinavelitonave. All 351 patients were discharged and the median hospital days was 14.5 ± 4.3 days.

**Table 3:** Demographic characteristics of 351 discharged patients with COVID-19.

	**Total patients (*n* = 351)**	**Re-positive (*n* = 17)**	**Negative (*n* = 334)**	***p***
Age, y	56.2 ± 15.8	53.8 ± 12.3	56.3 ± 16.0	0.507
≥60	164	6	158	0.465
<60	187	11	176	0.091
Gender, female/male	193/158	10/7	183/151	0.929
Relative diagnosed previously	44	4	40	0.629
Highest temperature, °C	37.8 ± 0.8	38.0 ± 0.7	37.8 ± 0.8	0.554
≥39.0	31	2	29	0.180
<39.0	320	15	305	0.889
Course of disease, d	14.6 ± 8.4	15.3 ± 7.9	14.5 ± 8.4	0.712
Hospital stay, d	14.5 ± 4.3	15.6 ± 4.7	14.4 ± 4.3	0.002
Clinical diagnose case	176	7	169	0.351
Classification Mild/common	104/209	7/9	97/200	0.276
Severe/critical	35/3	1/0	34/3	0.010
Under health conditions	177	6	171	0.086
Initial symptoms Fever	235	11	224	0.187
Respiratory	266	12	254	1.000
Digestive	31	3	28	0.231
Neuromuscular	150	9	141	0.063
Others	15	2	13	0.499
Lowest blood oxygen, %	95.1 ± 2.5	94.6 ± 3.9	95.1 ± 2.4	0.874
Oxygen inhalation Normal	165	8	157	>0.05
1–2 L	65	1	64	>0.05
2–4 L	31	0	31	>0.05
>4 L	87	7	80	>0.05
Mask	3	1	2	>0.05
Therapy Antiviral	233	11	222	0.229
Antibiotic	140	5	135	0.297
Hormone	18	0	18	0.231
Resochin	35	2	33	0.496
Traditional Chinese medicine	287	15	272	1.000
CT findings Mild	24	1	2	>0.05
Moderate	77	5	72	>0.05
Severe	225	8	217	>0.05
Blood routine examination				
WBC, *10^∧^9/L	5.8 ± 1.9	6.2 ± 2.1	5.8 ± 1.9	0.943
LYM, *10^∧^9/L	1.7 ± 0.6	1.7 ± 0.6	1.7 ± 0.6	0.670

*Re-positive, patients with re-detectable positive SRAS-CoV-2 RNA tests after hospital discharged*.

*Negative, patients with negative SRAS-CoV-2 RNA tests after hospital discharged*.

*Course of disease, the median days of illness onset to hospital*.

*CT, computerized tomography; WBC, white blood cell count; LYM, absolute lymphocyte count*.

Compared to patients with negative SARS-CoV-2 RNA test results after hospital discharge, patients with positive results seemed likely to have experienced less severe or critical illness (5.9 vs. 11.1%, *p* = 0.01). Although length of hospital stay showed a statistically significant difference between the two groups, the median length of hospital stay was similar between the groups (15.6 ± 4.7 vs. 14.4 ± 4.3 days, *p* < 0.01). There was no marked difference between the two groups in age, gender, history of close contact, highest temperature, lowest degree of blood oxygen saturation, course of disease, length of hospital stay, background disease, therapy strategies, CT findings or routine blood examination on admission. These results indicated that discharged patients with redetectable positive SARS-CoV-2 RNA results were difficult to predict. Therefore, it is an urgent requirement to find the high-risk factors associated with repositive patients.

### Prediction Model Benchmark

As a powerful predictive model to nonlinearly approximate statistical relationships in variables, the ensemble learning algorithm RF is implemented by the Random Forest Classifier package in Python 3.6, aiming to reliably identify the high-risk patients and quantify feature importance in function approximation. To guarantee a reliable classifier, it is essential to determine the optimal value of two hyperparameters, which are n_estimators and max_depth. To be more specific, the number of trees in the forest is defined as {10, 20, 30, 40, 50, 60}. The maximum depth of the tree, which controls the longest path between the root node and the leaf node, is fixed from 2 to 10 using the interval one. For hyperparameter tuning, the grid search under 5-fold cross validation runs an exhaustive search over all the 54 different RF prediction models, which will return the best model providing the highest average F1 score on the training set. Herein, the RF model will be developed using the chosen hyperparameter values: n_estimators = 50 and max_depth = 3.

From a model comparison experiment, the established RF model stands out with more outstanding classification performance than other popular machine learning models listed in Table 4, including k-nearest neighbors (KNN), Gaussian Bayesian (GB), logistic regression (LR), support vector machine (SVM), and AdaBoost. For reaching a more objective comparison, the grid search with 5-fold cross validation is also used to fine-tune these five comparison models. Except for GB, all models can provide dependable predations under the weighted F1 score >0.75. According to the comparison in the test set, RF performs the best in terms of all evaluation metrics, which is followed by SVM and AdaBoost. In regard to the result of label 1, the precision and F1 score from RF can be raised by at least 39.39 and 25.00%, indicating that RF is more capable to pick out these high-risk patients. Moreover, RF achieves the highest weighted F1 score of 0.85, which is 0.05 and 0.22 larger than second-best model SVM and the worst model GB, respectively. That is to say, RF can generate relatively high value for both precision and recall, which is a reasonable choice in this research to perform the best in distinguishing patients with high risk or not.

**Table 4:** Classification performance of other five popular machine learning models.

**Method**			**KNN**	**Gaussian Bayesian**	**Logistic regression**	**SVM**	**AdaBoost**
Parameter			n_neighbors = 2	var_smoothing = 1e-8	C = 0.1 Penalty = “L2”	kernel = “rbf” gamma = 0.0001 C = 200	n_estimator = 3 Learning rate = 1
Metric	Accuracy		0.75	0.58	0.76	0.79	0.76
	AUC		0.62	0.62	0.63	0.74	0.72
	Precision	Label = 0	0.91	0.92	0.91	0.94	0.94
		Label = 1	0.24	0.18	0.25	0.33	0.30
		Macro average	0.57	0.55	0.58	0.64	0.62
		Weighted Average	0.82	0.83	0.83	0.87	0.86
	Recall	Label = 0	0.79	0.56	0.81	0.81	0.77
		Label = 1	0.44	0.67	0.44	0.67	0.67
		Macro average	0.62	0.62	0.63	0.74	0.72
		Weighted average	0.75	0.58	0.76	0.79	0.76
	F1-score	Label = 0	0.84	0.70	0.85	0.87	0.85
		Label = 1	0.31	0.29	0.32	0.44	0.41
		Macro average	0.58	0.49	0.59	0.66	0.63
		Weighted average	0.78	0.65	0.79	0.82	0.79

### Prediction of High-Risk Patients

**Figure 3:**
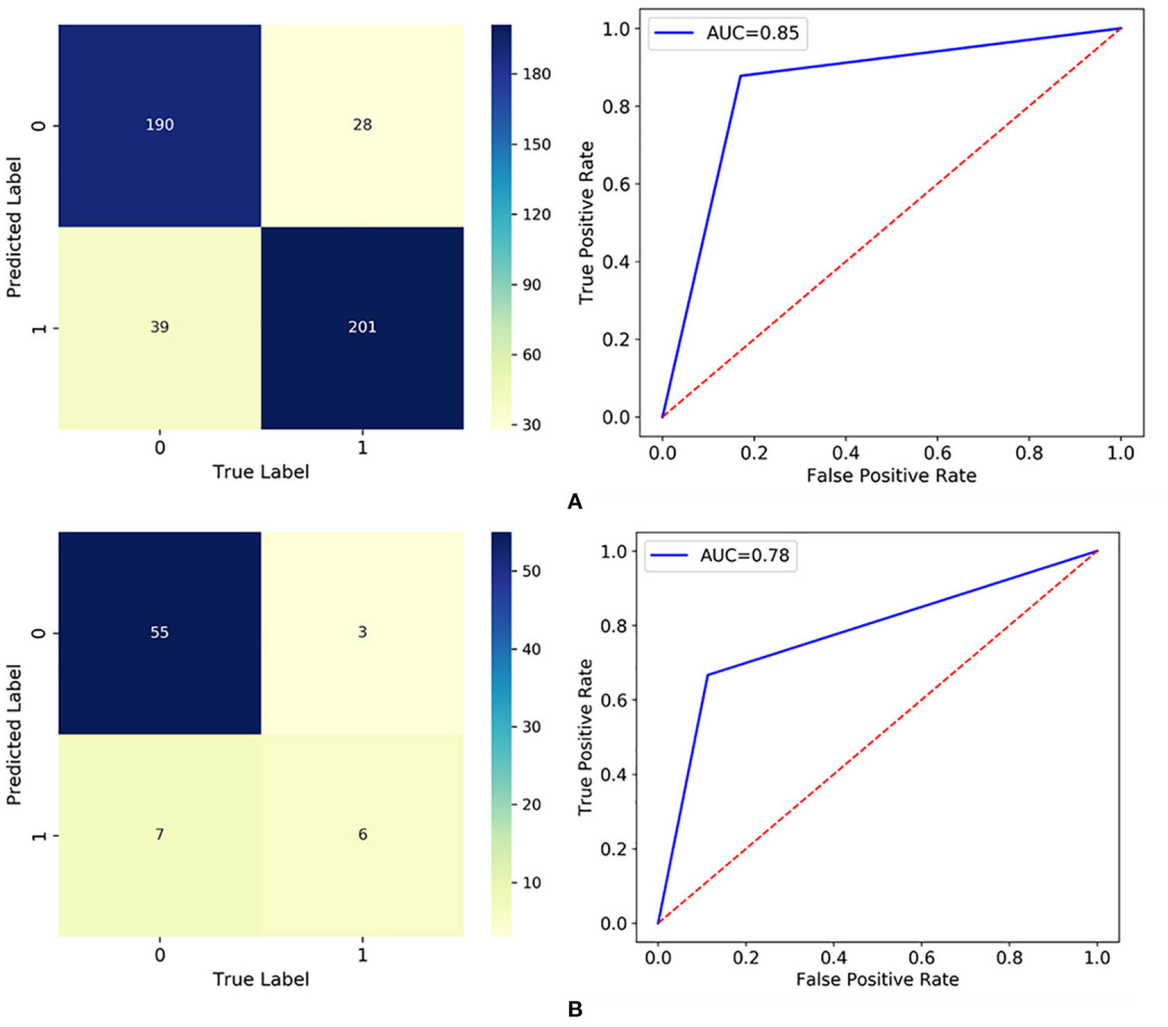
Classification results visualized by the confusion matrix and ROC curve in: **(A)** the training set; and **(B)** the test set.

The established RF model was then trained and tested on the two divided subsets, and its performance is summarized in Figure 3 and Table 5. In particular, the out of bag (OOB) score, which is calculated as the number of correctly predicted rows from the OOB sample, is 76.2%, indicating high generalization ability for the model. The training set and test set had almost the same accuracy and micro F1 score, confirming the approximate fitting of the model. Although the number of patients accurately assigned to label 1 in the test set was <85.5% of those accurately assigned to label 0, 66.7% (six out of nine) of high-risk patients could be correctly predicted as label 1, allowing them to receive additional attention. However, the precision in the test set (46.2%, six out of 13) was comparatively small, since seven low-risk patients (label 0) were mistakenly classified as label 1. As a trade-off between precision and recall, the average and weighted F1 scores could reach high values of 0.73 and 0.87, indicating promising classification performance for the developed RF model in successfully finding discharged patients with positive RT-PCR results and symptoms. Moreover, the ROC curve diverged from the 45-degree line near the coordinates (0, 0) and (1, 1) and yielded an AUC value of 0.78, which also indicates that the model has acceptable discrimination to diagnose patients with low and high risk.

**Table 5:** Summary of classification performance of RF model in the training and test set.

		**Precision**	**Recall**	**F1-score**	**Data size**
Train set	Label = 0	0.87	0.83	0.85	229
	Label = 1	0.84	0.88	0.86	229
	Macro average	0.85	0.85	0.85	
	Weighted average	0.85	0.85	0.85	
	Accuracy	0.85			
Test set	Label = 0	0.95	0.89	0.92	62
	Label = 1	0.46	0.67	0.55	9
	Macro average	0.70	0.78	0.73	
	Weighted average	0.89	0.86	0.87	
	Accuracy	0.86			

### Discovery of High-Risk Factors

**Figure 4:**
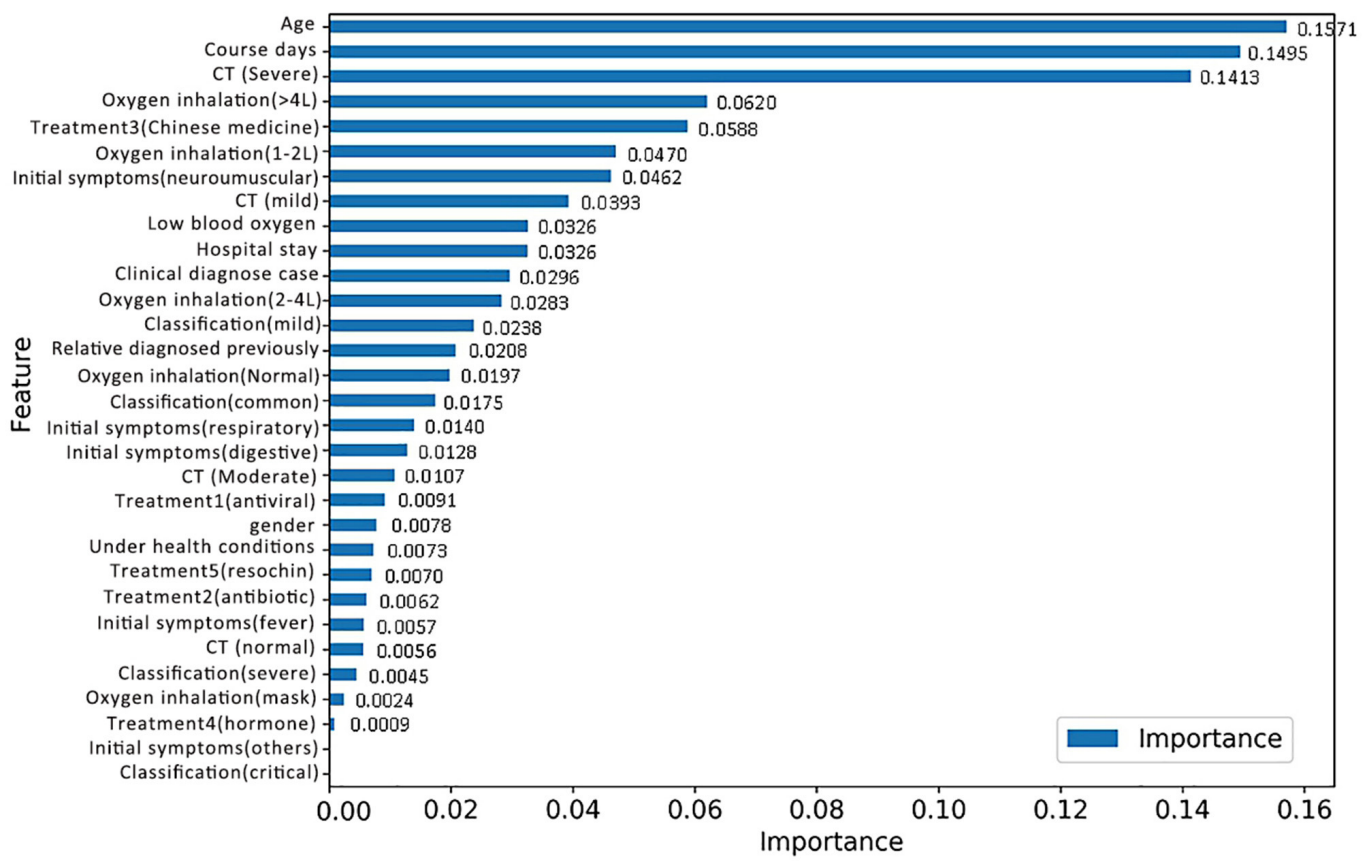
Feature importance based on the RF model for discharged patients with positive RT-PCR test results and symptoms.

**Figure 5:**
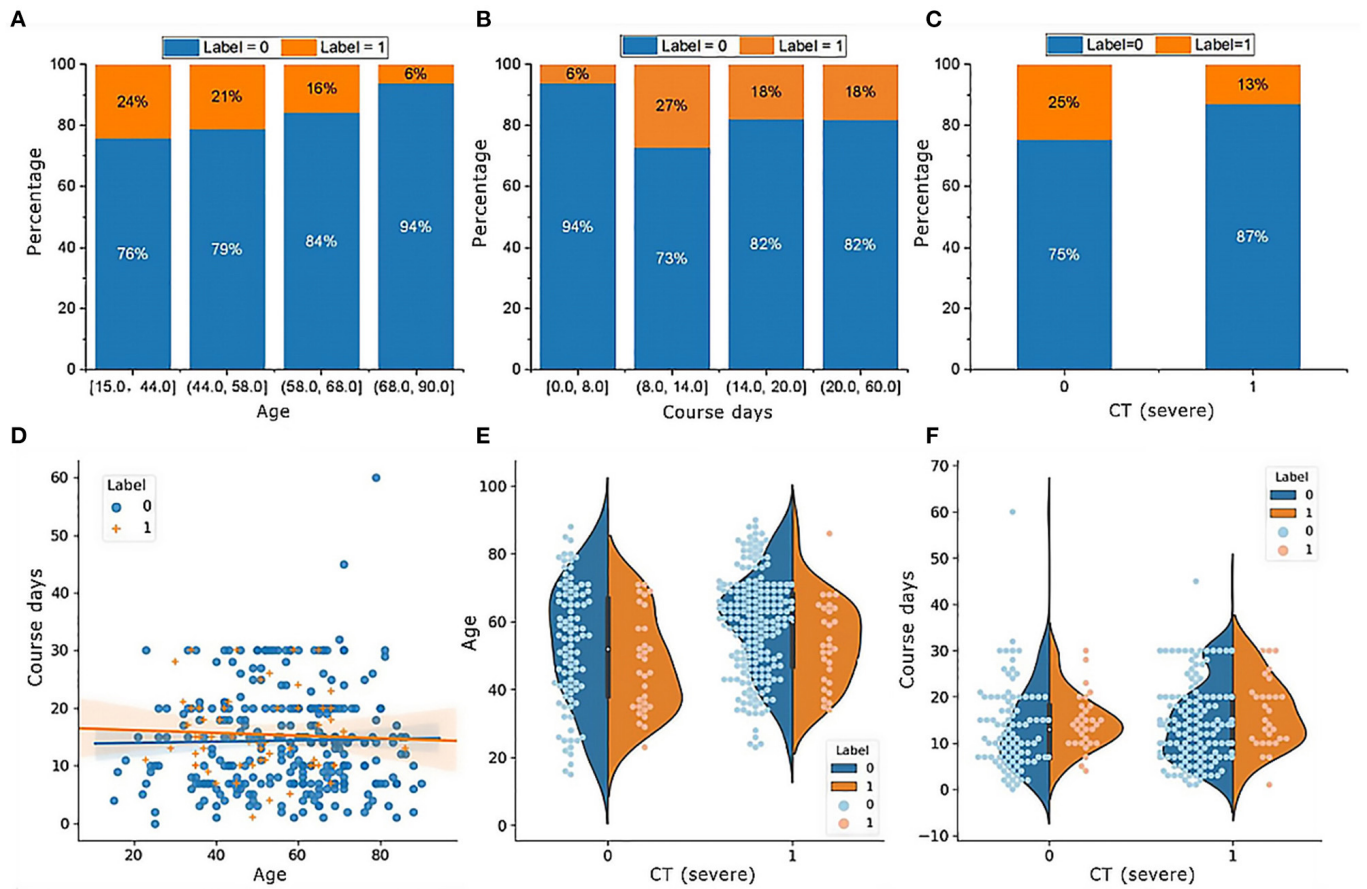
Characteristics of the top three important features in patients with or without positive RT-PCR test results and symptoms after hospital discharge. **(A)** The percentage of patients in label 1 gradually decreases with increasing age; **(B)** patients with label 1 account for the smallest portion (6%) of the total patients; **(C)** patients with label 1 in the group of CT (severe) is 12% smaller than CT (nonsevere); **(D)** there is no distinct rule in the course of disease for different ages of patients; **(E)** for patients in the group of CT (nonsevere), the age in label 1 tends to be smaller than label 0, while their course of disease could be longer in **(F)**.

**Figure 6:**
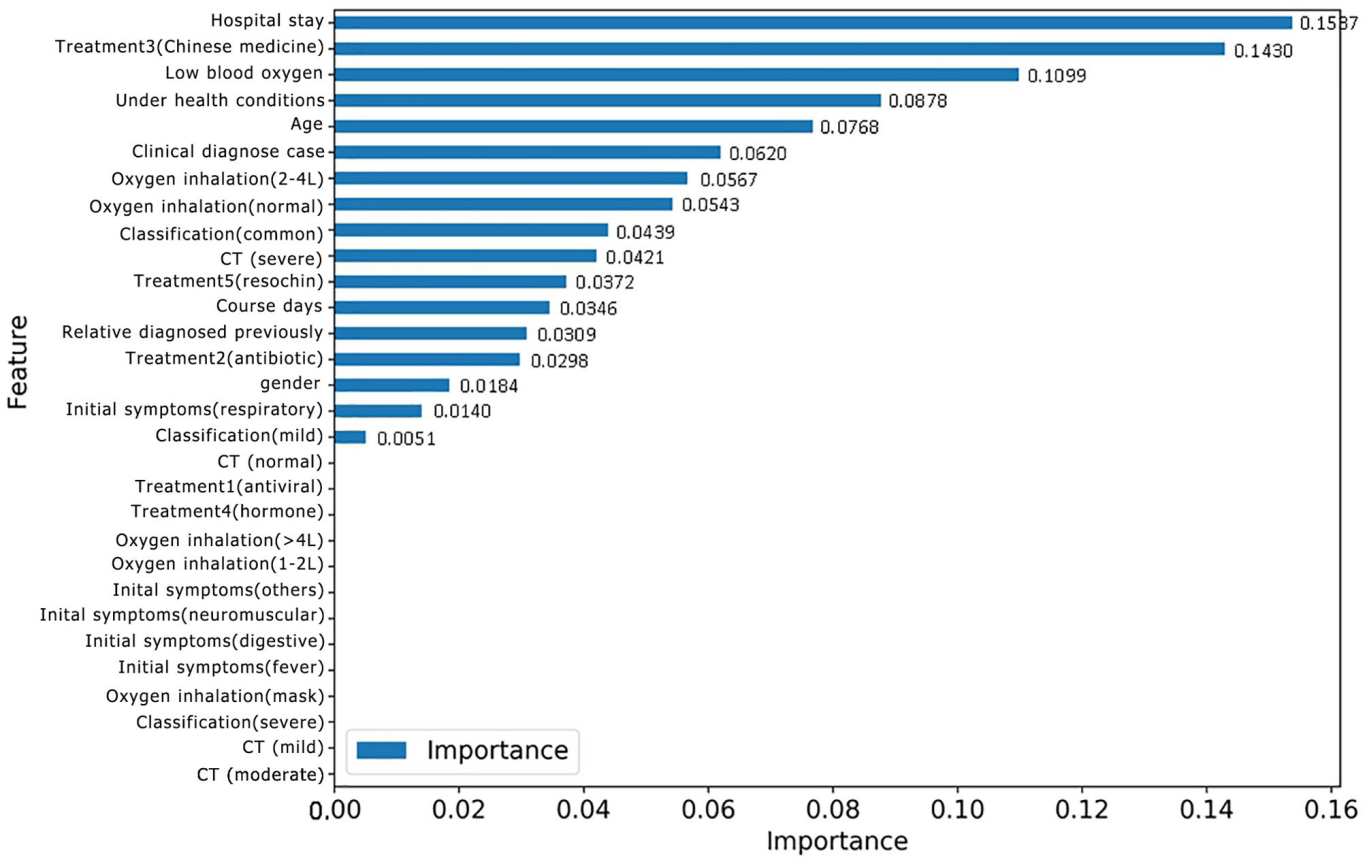
Feature importance based on the new RF model for classifying patients with or without positive RT-PCR after leaving hospital.

The RF model allows the quantification and explanation of the roles of input features in classification performance, which are described in Figure 4. It can be observed that the RF model makes decisions mostly depending on the features age, course of disease, and CT (severe), whose total feature importance makes up nearly half (44.79%) of the total. A better understanding of the relationship between the classification results and the top three most influential features is presented in Figure 6. Specifically, the percentage of patients in label 1 gradually decreased with increasing age (Figure 5A), which suggests that patients at ages younger than the middle quantile (50%) were more likely to have positive RT-PCR results and symptoms. As shown in Figure 5B, patients with label 1 accounted for the smallest portion (6%) of the total patients in the first (25%) quantile, and thus, patients who experienced a course of disease shorter than 8 days were more likely to completely recover. As shown in Figure 5C, the number of patients with label 1 in the severe CT group was 12% lower than that in the nonsevere CT group, which implied that patients with nonsevere CT conditions rather than severe CT conditions should receive more attention after recovery. Additional correlations among these three important features are described below. There was no distinct rule in the course of disease (short or long) for different patient ages (Figure 5D). As shown by the violin plot in Figure 5E, high-risk patients with severe CT appeared to be younger than those with nonsevere CT. For patients in the nonsevere CT group, the patients in label 1 tended to be younger than those in label 0 (Figure 5E), while their course of disease could be longer (Figure 5F). Conversely, for patients in the severe CT group, no significant difference existed between the two classes in terms of age and disease course.

### Identifying Discharged Patients With Positive RT-PCR Results

For the purpose of identifying repositive patients among all the high-risk patients, records with the label 1 were separated from the overall dataset and then fed into a new RF model. First, data labeling was performed, and new labels called positive 0 and positive 1, which refer to patients with symptoms and positive nucleic acid test results, respectively, were assigned to 43 and 17 records. Afterward, the training and testing process of the new RF model was repeated, resulting in the classification performance shown in Table 6. Although patients with symptoms tended to be falsely identified as repositive patients (precision 0.43), all patients with positive RT-PCR tests in the dataset could be predicted correctly (recall 1.0). That is, this model, with a weighted F1 score of 0.69, allowed reliable recognition of patients with positive RT-PCR results at an early stage, which was one of the key goals of this research. Figure 6 shows the corresponding feature importance in descending order. It is clear that only the 56.67% of features (17 out of 30) with feature importance values >0 were truly in play. Here, the three features hospital stay (0.1537), treatment 3 (Chinese medicine) (0.1430), and low blood oxygen (0.1099) contributed more to classifying high-risk patients with positive nucleic acid test results.

**Table 6:** Summary of classification performance of the new RF model in classifying patients with or without positive RT-PCR tests.

	**Precision**	**Recall**	**F1-Score**
Positive = 0	1.00	0.56	0.71
Positive = 1	0.43	1.00	0.6
Macro average	0.71	0.78	0.66
Weighted average	0.86	0.67	0.69
Accuracy	0.67		
AUC	0.78		

**Figure 7:**
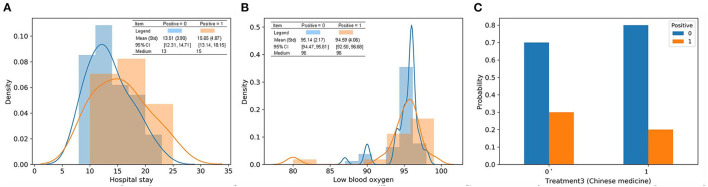
Characteristics of the top three most important features in discharged patients with or without positive RT-PCR after leaving hospital. **(A)** Patients in positive 1 tend to stay in the hospital for an extra 2 days on average from the distribution; **(B)** the range of lowest blood oxygen for patients in positive 1 is wider than positive 0; **(C)** 20% patients using treatment 3 had positive RT-PCR tests, which is 10% less than patients in the group without treatment 3.

The characteristics of the three most important features in the two classes are visualized in Figure 7. Based on the distribution, positive 1 patients tended to stay in the hospital for an additional two days on average. The range of low blood oxygen for positive 1 patients was wider than that for positive 0 patients, indicating a higher level of uncertainty. These results indicated that a long hospital stay was more likely to be associated with repositive patients. In particular, the median hospital stay in 11 out of 17 (64.7%) repositive patients was more than 15 days, while only 15 of 43 (34.9%) patients had negative RT-PCR results. The standard deviation of blood oxygen saturation in patients with positive RT-PCR results was larger than that in patients with negative RT-PCR results, indicating that the blood oxygen saturation of repositive patients had a wider distribution and greater uncertainty. Remarkably, only 20% of patients using Chinese medicine were repositive, which was 10% less than the patients in the group with no Chinese medicine. It can be concluded that traditional Chinese medicine can possibly reduce the repositive rate to some extent. The effect of traditional Chinese medicine remains to be further studied.

## Discussion

We reported 406 patients discharged from Leishenshan Hospital in Wuhan, China between February 8 and March 8, 2020, in this study. Nineteen (4.67%) patients had positive RT-PCR test results 5 to 14 days later. Current available evidence suggests that several reasons contributed to the phenomenon of recovered patients with redetectable positive RT-PCR test results, including (a) genetic material contamination; (b) the use of different commercial kits; (c) sampling error involving throat swabs; (d) virus remaining in discharged patients; and (e) possible intermittent detoxification in some discharged patients (11–17). In addition, the quality of the sample, the method of sampling and the expertise level of the sample collector also influence real-time RT-PCR test results (18). In the present study, two discharged patients had three repeat positive nucleic acid tests over the next 2–7 days. This suggests that a proportion of recovered patients may still be likely to become SARS-CoV-2 carriers, not false positives, which is consistent with previous findings (19).

The clinical characteristics, laboratory findings, and radiological features of recovered patients with or without positive virus RNA tests were further compared, and no significant difference was found between them. Therefore, a RF model was employed to investigate the risk factors for positive RT-PCR test results in recovered patients. As a powerful predictive model to nonlinearly approximate statistical relationships in variables, RF was implemented in Python 3.6 to reliably identify high-risk patients and quantify feature importance in function approximation. Specifically, RF, a type of ensemble learning, combines multiple weak decision trees into a strong model; its advantages largely lie in handling high-dimensional and nonlinear variables and making precise classifications with little overfitting and easy implementation (6–8). Moreover, RF can provide a good indicator of feature importance to examine the causes and effects behind the input and output variables, making it preferable to uninterpretable black-box algorithms (9).

However, it is impossible to distinguish patients with high or low risk based on a small sample. To increase the number of positive samples, patients with positive RT-PCR results and symptoms after leaving the hospital were first defined as a high-risk population to improve the prediction accuracy of the RF model. In addition, the most important features could be used to identify patients with high potential for repositive nucleic acid tests. Subsequently, the data of patients who had positive RT-PCR results were separated from the high-risk population and added to the RF model training data. Finally, the feature importance of patients with positive RT-PCR results could be accurately distinguished.

Our study showed that length of hospital stay, blood oxygen level, and Chinese medicine treatment were closely correlated with positive SARS-CoV-2 RNA test results after release. Further studies implied that patients with the highest (≥97%) and lowest (≤85%) blood oxygen saturation levels were more likely to have positive RT-PCR results; most patient blood oxygen values were concentrated at approximately 95 and 96%. At present, no definite conclusion has been reached about whether recovered patients with positive nucleic acid results are still infectious. In the cohort series, the longest time after discharge for patients with a first positive RT-PCR test was 14 days. Hence, patients with the above high-risk factors should be supported, managed and isolated for at least 14 days after hospital discharge. Meanwhile, the criteria for leaving the hospital or discontinuation of quarantine and continued patient management should be reevaluated. Increasing the number of nucleic acid tests or multisite sampling (nasopharyngeal swabs, sputum swabs or anal swabs) can be considered as new criteria for hospital discharge.

This study has several limitations. Our study was a single-center retrospective analysis, and only 351 patients discharged from the hospital were included. Some specific information regarding SARS-CoV-2 RNA test results was lacking (rectal, anal, or nasopharyngeal swabs) owing to the limited conditions. Moreover, because of a lack of data, the serum levels of specific antibodies and other laboratory findings in discharged patients were not presented. In addition, the epidemiology, demography, clinical characteristics, detailed laboratory indices, radiographic results, and follow-up information of the redetectable patients discharged from the hospital were not comprehensively compared with those of the remaining patients. A long-term observation and prospective study design would help to define the potential high-risk factors of repositive patients and to investigate the underlying mechanism.

## Data Availability Statement

The original contributions presented in the study are included in the article/supplementary material, further inquiries can be directed to the corresponding authors.

## Ethics Statement

The studies involving human participants were reviewed and approved by the Ethics Committee of Zhongnan Hospital of Wuhan University. Written informed consent to participate in this study was provided by the participants' legal guardian/next of kin.

## Author Contributions

YQ, GZ, and YP: conceived and designed the study. GZ: contributed to the literature search. YP: contributed to data collection. LZ, KL, and YL: contributed to data analysis. KL: contributed to data interpretation. YQ, YL, and GZ: contributed to writing of this article. All authors contributed to the article and approved the submitted version.

## Conflict of Interest

The authors declare that the research was conducted in the absence of any commercial or financial relationships that could be construed as a potential conflict of interest.

## Publisher's Note

All claims expressed in this article are solely those of the authors and do not necessarily represent those of their affiliated organizations, or those of the publisher, the editors and the reviewers. Any product that may be evaluated in this article, or claim that may be made by its manufacturer, is not guaranteed or endorsed by the publisher.
